# Analysis of the torque capacity of a completely customized lingual appliance of the next generation

**DOI:** 10.1186/1746-160X-10-4

**Published:** 2014-02-07

**Authors:** Stefan Lossdörfer, Carsten Bieber, Rainer Schwestka-Polly, Dirk Wiechmann

**Affiliations:** 1 Department of Orthodontics, Dental Clinic, University of Bonn, Welschnonnenstr 17, Bonn 53111, Germany; 2 Orthodontic practice, Arnhofener Str. 4 ½, Aindling 86447, Germany; 3 Orthodontic practice, Beethovenstr. 8, Leipzig 04107, Germany; 4 Department of Orthodontics, Hannover Medical School, Carl-Neuberg-Str. 1, Hannover 30625, Germany; 5 Orthodontic practice, Lindenstr. 44, Bad Essen 49152, Germany

**Keywords:** Torque angle, Torque moment, Completely customized lingual appliance

## Abstract

**Introduction:**

In lingual orthodontic therapy, effective torque control of the incisors is crucial due to the biomechanical particularities associated with the point of force application and the tight link between third order deviations and vertical tooth position.

**Aim:**

The aim of the present *in vitro* investigation was to analyze the torque capacity of a completely customized lingual appliance of the next generation (WIN) in combination with different finishing archwire dimensions.

**Methods:**

Using a typodont of the upper arch carrying the WIN appliance, slot filling and undersized individualized β-titanium archwires were engaged. Horizontal forces ranging from 0 to 100 cN were applied at the central incisor by means of spring gauges. The resulting angular deviations were recorded and the corresponding torque moments were calculated.

**Results:**

For fullsize archwires (0.018”×0.018” β-titanium and 0.018”×0.025” β-titanium), an initial torque play of 0-2° had to be overcome prior to the development of an effective torque moment. Thereafter, a linear correlation between torque angle and torque moment developed for both archwire dimensions with steeper slopes calculated for the specimens with the larger dimension. A torque moment of 2 Nmm required for effective torque correction was noted after a minimum of 2-3° of twist for the 0.018”×0.018” β-titanium wires as compared to 2-4° for the 0.018”×0.025” β-titanium study sample. When undersized archwires were analyzed (0.0175”×0.0175” β-titanium), the measured torque play ranged from 5-7°. After 8-12° of torque angle, the threshold of 2 Nmm was reached. A linear relationship between twist angle and torque moment in which the steepness of the slopes was generally flatter than the ones calculated for the slot filling archwires was noted.

**Conclusions:**

Given the high precision of the bracket slot-archwire-combination provided with the WIN appliance, an effective torque control can be clinically realized.

## Introduction

Fixed orthodontic appliances allow for three-dimensional control of teeth during complex tooth movements; these include bodily movements, intrusion or extrusion, and torque correction requiring a change in tooth root but not crown position [1].

Among different choices, completely customized lingual appliances (CCLA) represent an alternative that has certain advantages over non-customized appliances bonded to the labial surface of the teeth. Other than the aesthetic aspects that meet the increasing demand for invisible appliances, potential benefits include the accuracy of the treatment outcome resulting from the sophisticated laboratory process that involves a target set-up [2] and from the precision of the bracket slot-archwire-combination [3,4]. Furthermore, a reduced risk of decalcification was reported [5]. Lingual appliances exhibit some biomechanical particularities due to the different point of force application and the resulting line of force relative to the center of resistance of the tooth [6], as well as from a shorter inter-bracket distance with the respective consequences for free archwire length and wire stiffness. The ability of an appliance to transfer a certain torque movement depends on complex interactions of several parameters including bracket material and deformability, archwire alloy and dimension, type of ligature, and torsion and play of the archwire [7-15].

Effective torque control of the incisors is of particular importance in lingual orthodontics because there is a tight link between third order problems and the vertical position of the tooth [16]. With increasing distance of the point of force application from the labial tooth surface, any deviations in torque will result in more evident variations in vertical tooth position [17]. Additionally, the distance between the point of force application and the center of resistance might be different in lingual and labial appliances [18]. Hence, torque control is more difficult, but crucial in lingual orthodontics [6,19]. The reduction in thickness of the lingual appliance and in positioning thickness constituted a major developmental progress not only in terms of patient comfort, but also in clinical performance of the appliance. This minimized the need for corrective bends during the finishing phase of an orthodontic treatment [20].

Apart from these considerations, torque control is of further significance in the effort to preserve the periodontal integrity of the dentoalveolar complex during tooth movement. There is evidence that certain types of tooth movement bear a higher risk for the development of bony dehiscences, fenestrations, and gingival recessions than others depending on the morphology of the tooth supporting apparatus, including soft and hard tissues. For example, Sperry *et al*. pointed to an increased incidence of labial recessions in cases of dentoalveolar compensation of class III malocclusion [21]. Their findings are supported by the observation that pre-treatment existent recessions and the retroclination of teeth in cases of mesiobasal relation constitute key risk factors for the development of further recessions [22]. Therefore, careful treatment planning and consideration of all relevant parameters and the subsequent transfer of the anticipated tooth position to the clinical situation are crucial for a successful treatment outcome.

**Figure 1:**
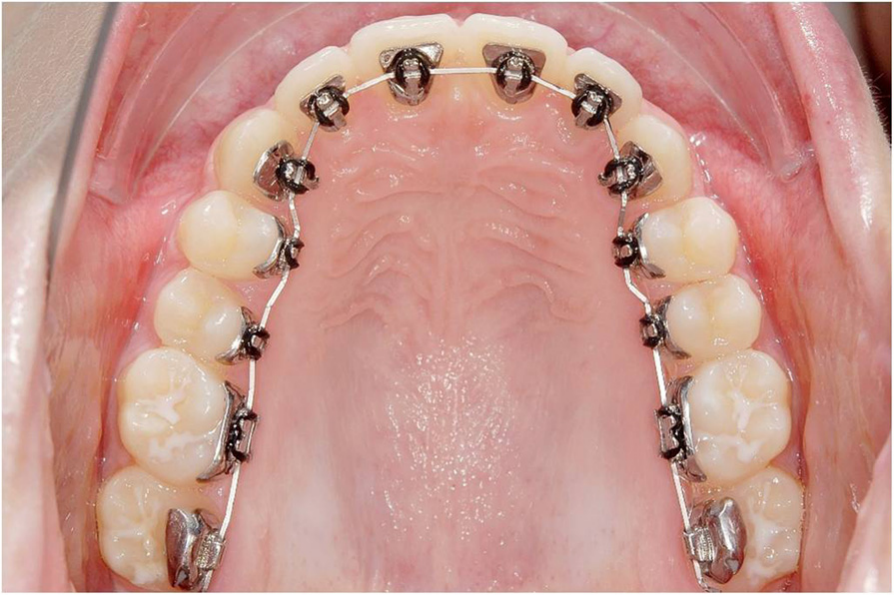
Completely customized lingual appliance of the next generation (WIN) bonded in the upper arch.

The aim of the present *in vitro* study was to analyze the torque capacity of a CCLA of the next generation (WIN; Figure 1) in combination with different archwire dimensions that are routinely used for finishing in lingual orthodontics. It was hypothesized that the initial torque play would be dependent on wire dimension. After a certain twist characteristic of archwire dimension, a linear correlation between torque angle and the resulting torque moment was assumed.

## Materials and methods

### Slot size

One of the determining factors for effective torque control is the precision of the bracket slot and archwire combination. The accuracy of the WIN bracket slot was recently demonstrated to correspond with the stated value of 0.0180” [23]. Those findings were taken as a basis for the interpretation of the results obtained in the present study.

### Archwire material and dimensions

**Figure 2:**
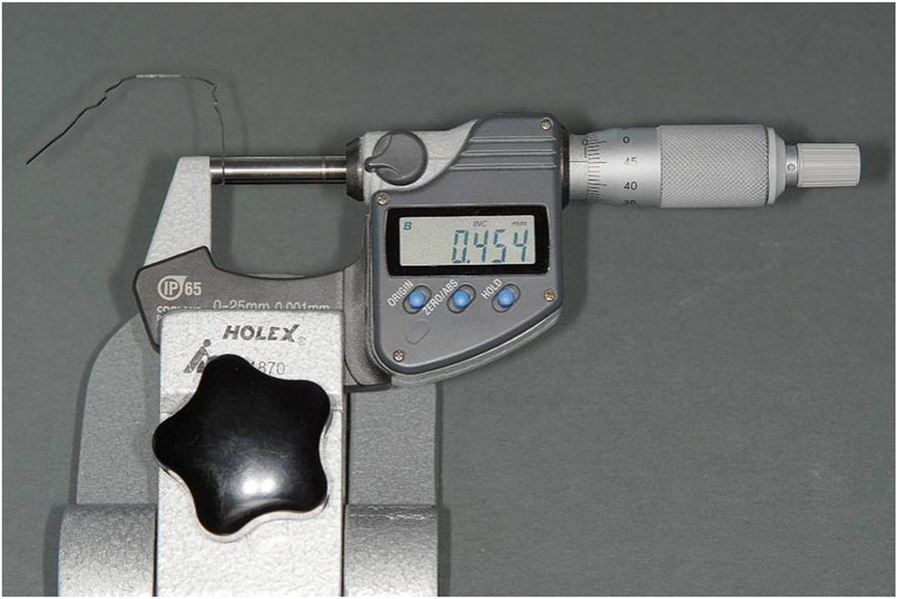
Digital caliper for the measurement of the archwire dimension.

The precision of the horizontal dimension of the two commonly used individualized finishing archwires (0.018”×0.018” β-titanium, 0.018”×0.025” β-titanium) was analyzed by a digital caliper allowing for accurate measurements in the range of 1 μm (Holex, Hoffmann Quality Tools, Munich, Germany) (Figure 2). For comparison purposes, 0.0175”×0.0175” β-titanium archwires were included as a reference for undersized dimension. Ten archwires of each slot filling size were measured in three different areas of the wire to ensure the representative character of the data obtained. Likewise, ten of the 0.0175×0.0175” β-titanium archwires were analyzed, since this wire dimension represents an often recommended finishing archwire in lingual orthodontics and was subject to analysis in several studies on torque capabilities [18].

### Torque capacity

**Figure 3:**
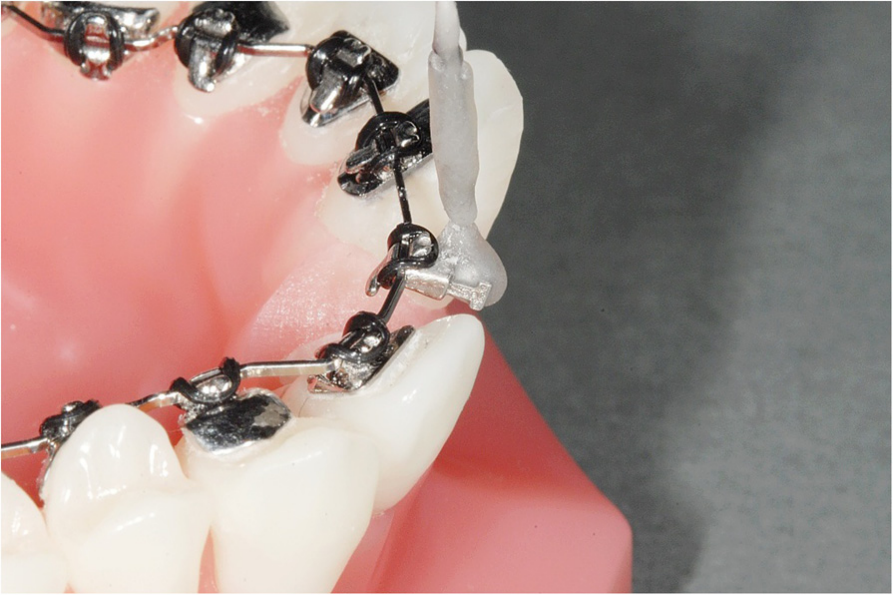
Typodont model carrying the WIN appliance with the central upper left incisor separated and the vertical extension for insertion of a spring gauge in situ.

**Figure 4:**
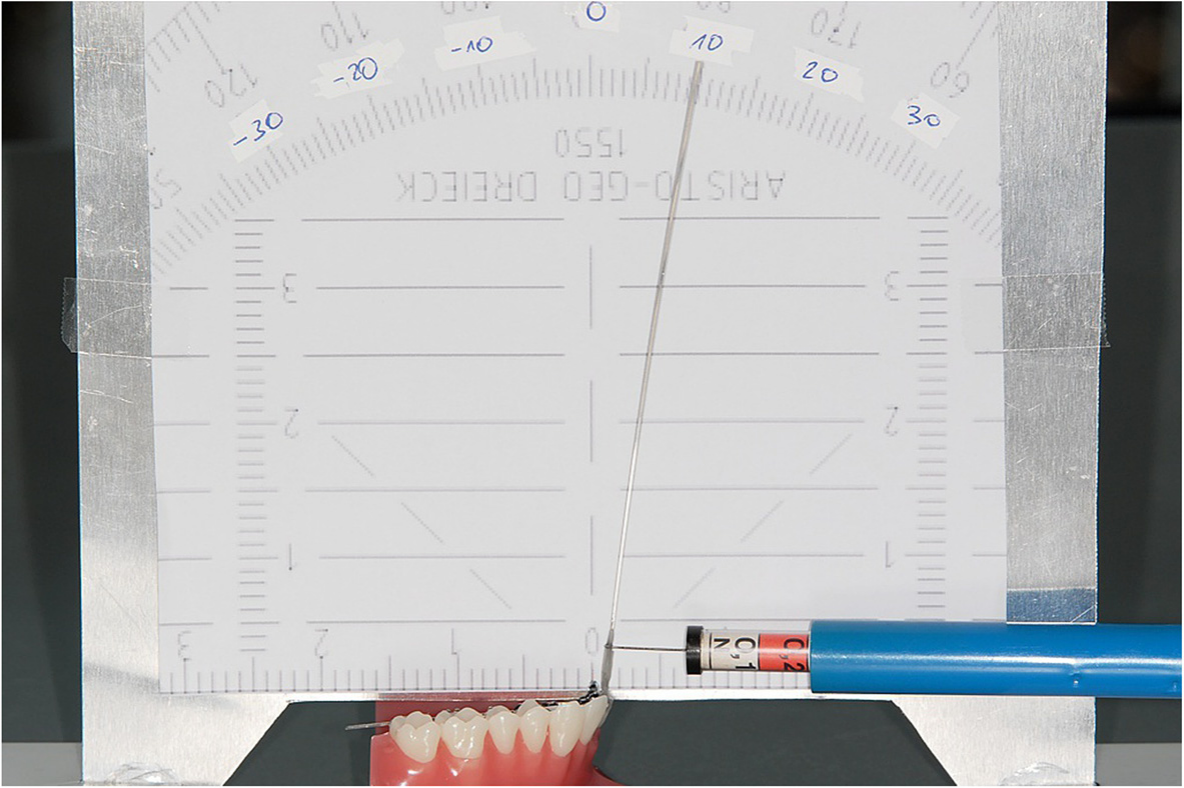
Typodont model in front of the measuring wall with a spring gauge inserted.

On a typodont carrying the WIN appliance (DW Lingual Systems GmbH, Bad Essen, Germany), the upper left central incisor was separated to allow for torque movements on the respective archwire which was ligated with alastics. A piece of wire extending vertically from the bracket body had an indentation at a 10 mm distance from the bracket slot serving as a defined reference for the insertion of commercially available spring gauges. These displayed either a tenth or hundredth part scaling ranging from 0 to 1 N (Figure 3). A measuring wall displaying a scale in degrees was attached to the typodont. Based on this experimental set-up, horizontal forces were applied by the respective spring gauge in deci steps in both directions representing positive and negative torque movements. The resulting angular excursions were recorded from the measuring wall (Figure 4). The applied torque moments, calculated by multiplying the horizontal force times the distance from the bracket slot, could be linked to the corresponding angular deviation.

### Repetitive measurements

Ten specimens of each slot filling archwires with a dimension of 0.018”×0.018” β-titanium and 0.018”×0.025” β-titanium were engaged in the typodont appliance and the angular excursions induced by the different forces were recorded as described above. The same procedure was carried out for ten 0.0175”×0.0175” β-titanium wires for comparison purposes.

## Results

### Archwire dimension

The dimension for the 0.018”×0.018” β-titanium archwires ranged from 0.01811” to 0.01827” equaling to 0.460 mm to 0.464 mm (Table 1) as opposed to a slight undersizing of the 0.018”×0.025” β-titanium archwires with measurements between 0.01776” to 0.01791” corresponding to 0.451 mm to 0.455 mm (Table 2). For comparison, the dimensional precision of the 0.0175”×0.0175” β-titanium revealed a slight undersizing with value obtained between 0.01732” to 0.01744” (0.440 mm to 0.443 mm) (Table 3).

**Table 1:** Dimensional variation of ten 0.018”×0.018” β-titanium archwires as determined by means of a digital caliper

**0.018”×0.018” β-titanium**	**1**	**2**	**3**	**4**	**5**	**6**	**7**	**8**	**9**	**10**
Dimension (mm)	0.462-0.463	0.462-0.463	0.460-0.462	0.460-0.462	0.461-0.464	0.461-0.464	0.461-0.464	0.461-0.464	0.460-0.462	0.460-0.462

**Table 2:** Variation of the horizontal dimension of ten 0.018”×0.025” β-titanium archwires as determined by means of a digital caliper

**0.018”×0.025” β-titanium**	**1**	**2**	**3**	**4**	**5**	**6**	**7**	**8**	**9**	**10**
Dimension (mm)	0.452-0.453	0.452-0.453	0.451-0.453	0.451-0.453	0.453-0.454	0.453-0.454	0.452-0.453	0.452-0.453	0.453-0.455	0.453-0.455

**Table 3:** Determination of the dimensional variation for ten archwires made of 0.0175”×0.0175” β-titanium serving as a reference for undersized wires

**0.0175”×0.0175”β-titanium**	**1**	**2**	**3**	**4**	**5**	**6**	**7**	**8**	**9**	**10**
Dimension (mm)	0.442-0.443	0.440-0.442	0.439-0.441	0.441-0.442	0.441-0.442	0.440-0.442	0.441-0.442	0.441-0.442	0.442-0.442	0.442-0.442

### Torque capacity

**Figure 5:**
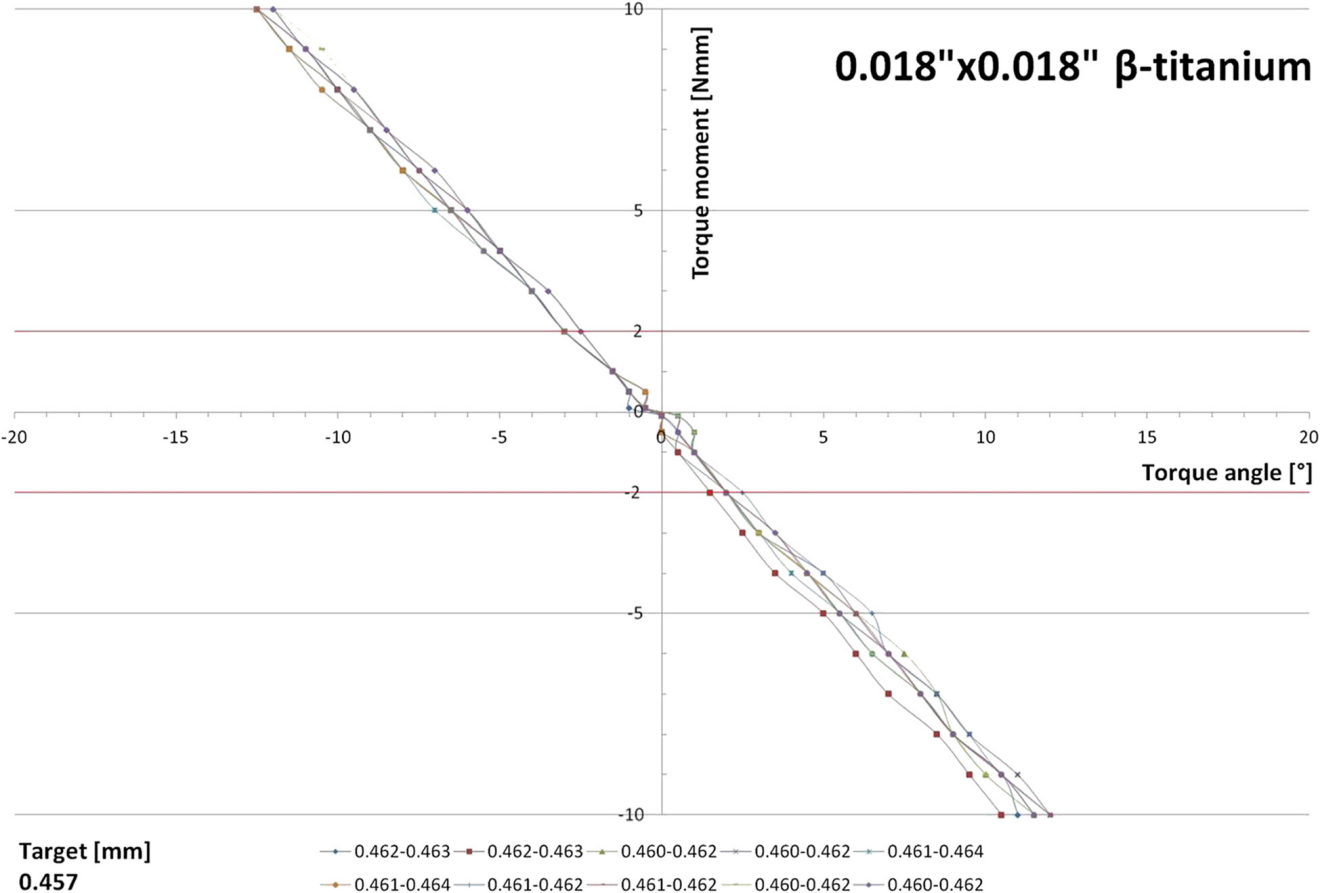
Torque moment characteristics of ten 0.018”×0.018” β-titanium archwires.

**Figure 6:**
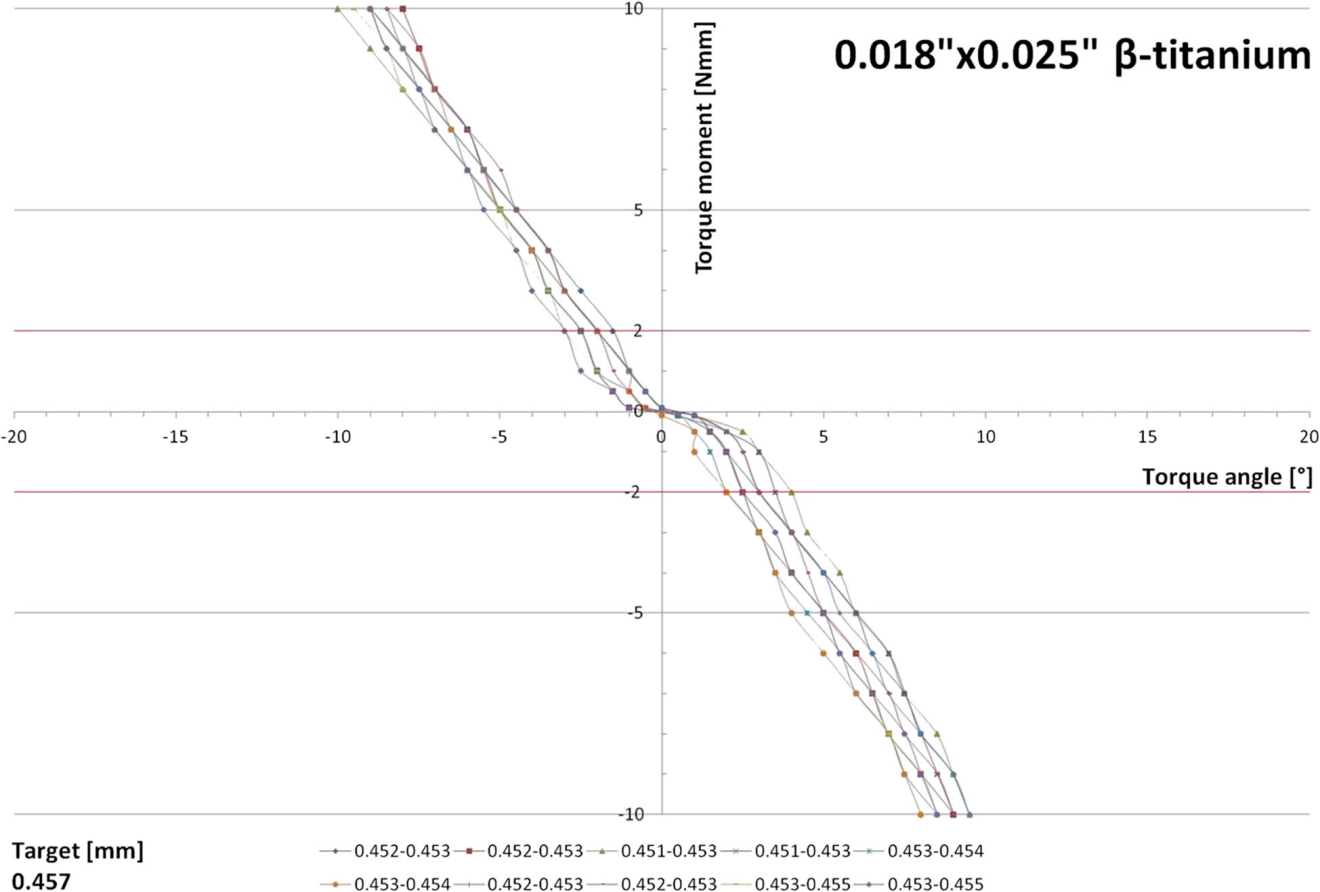
Correlation of torque angle and torque moment determined for ten 0.018”×0.025” β-titanium archwires.

When the 0.018”×0.018” β-titanium archwires were engaged in the typodont and horizontal forces were applied, similar results were obtained in all ten specimens examined. Lacking an initial torque play, a linear correlation between twist angle and torque moment developed. The threshold of 2 Nmm required for effective torque movement was reached after 2-3° of twist (Figure 5). A small torque play was recorded for the 0.018”×0.025” β-titanium archwires (1-2°); 2 Nmm of torque moment were reached after an angular deviation of 2-4° and steeper slopes of the torque angle/torque moment ratio were calculated for this wire dimension (Figure 6).

**Figure 7:**
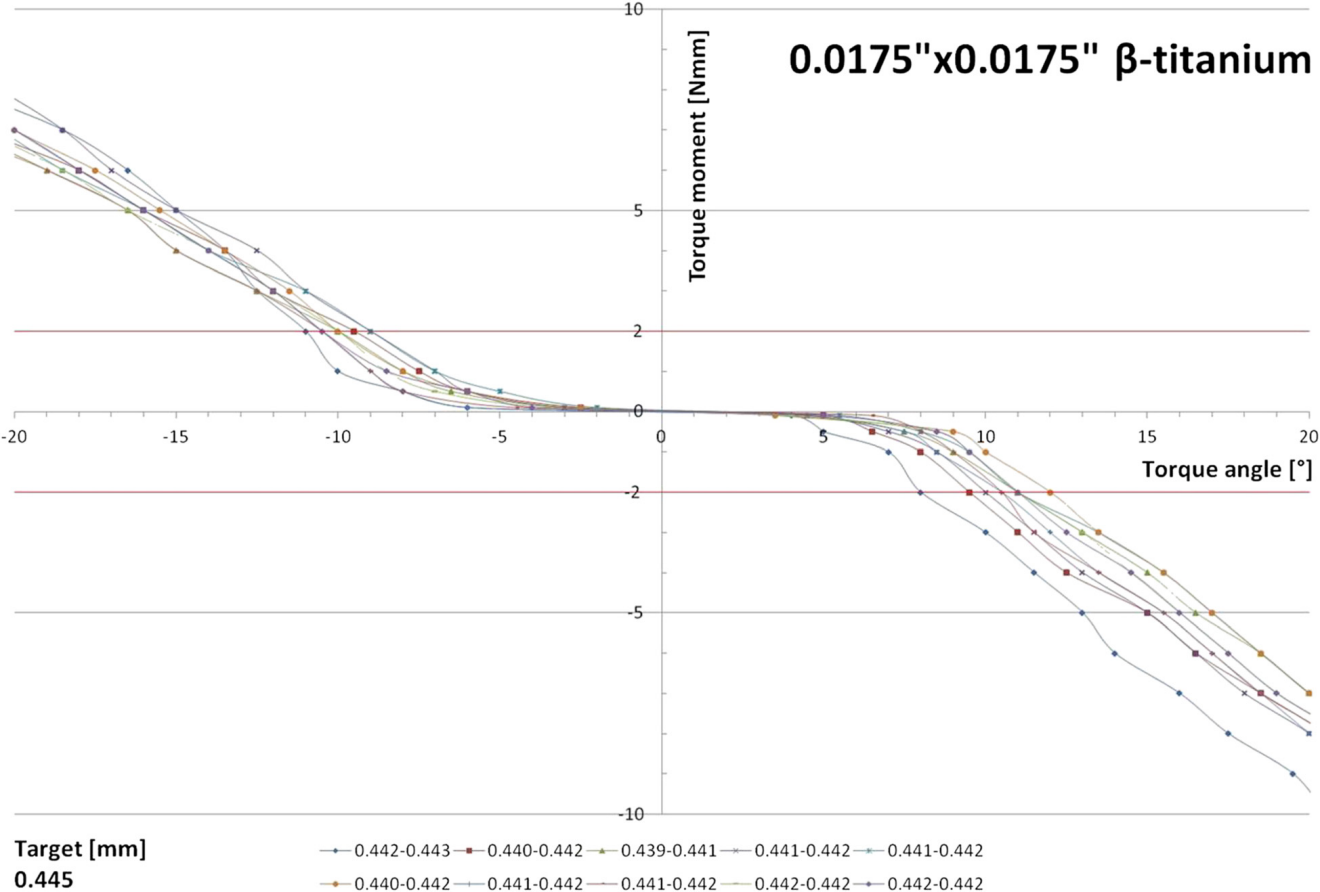
Torque moment characteristics of ten 0.0175”×0.0175” β-titanium archwires.

Regarding the undersized reference archwires, 5-7° of torque play were characteristic of the 0.0175”×0.0175” β-titanium specimens. Once torque play was overcome, a linear curve shape developed, with a flatter slope and requiring a minimum of 8-12° of torque angle for the development of 2 Nmm of torque moment (Figure 7).

**Figure 8:**
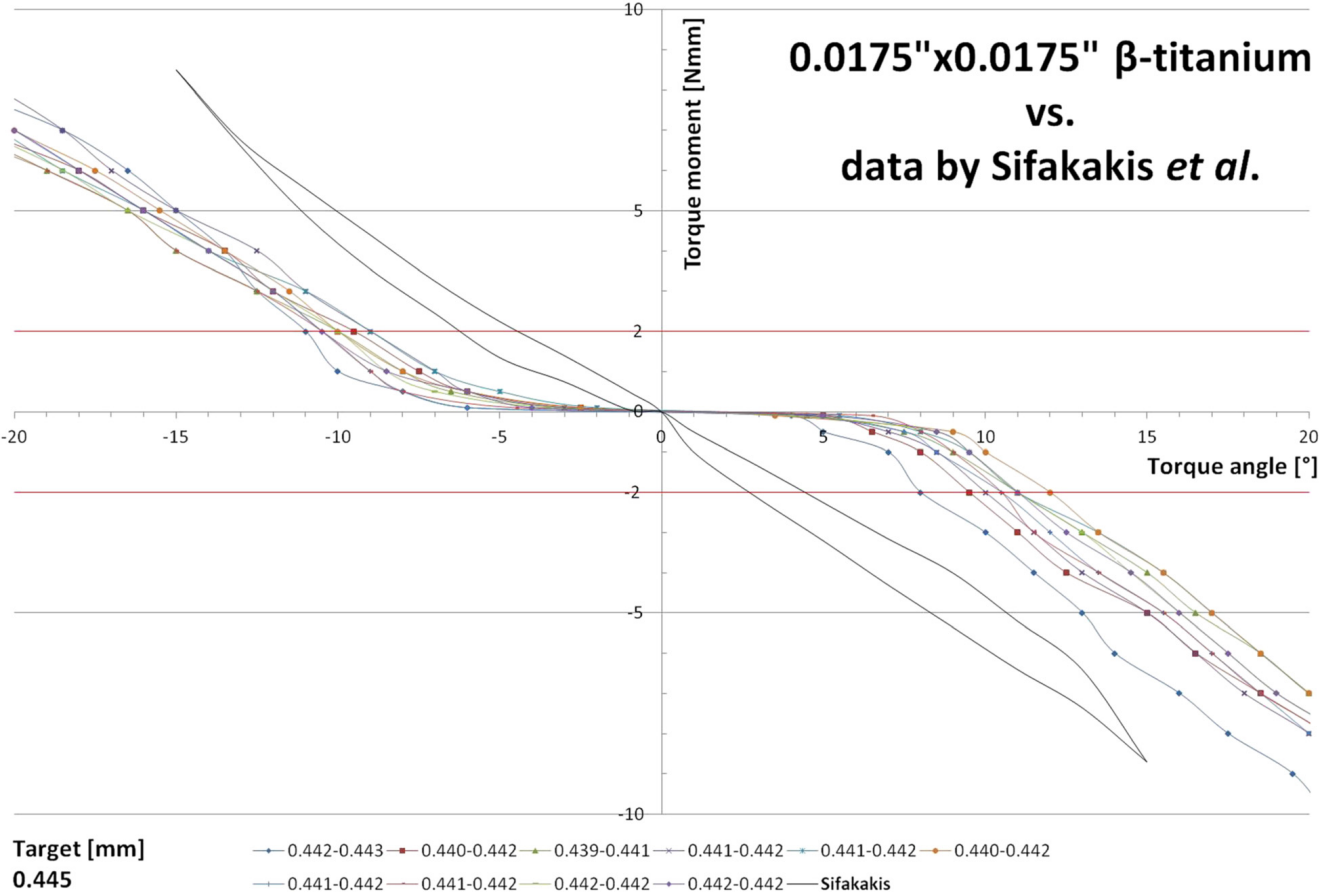
**Comparison of the torque characteristics of the 0.0175”×0.0175” β-titanium archwires analyzed in the present study (lines marked with squares) vs. the results presented by Sifakakis et al. [**[18]**] examining the same archwire dimension and material in an Incognito™ appliance.**

Figure 8 compares the torque characteristics of the 0.0175”×0.0175” β-titanium archwires included in the present study to recently published material examining the same archwire material and dimension in a different experimental set-up [18]. In contrast to our results, the reference data did not yield any torque play for this undersized archwire and presents with a generally steeper slope.

## Discussion

The present *in vitro* experiment examined the torque characteristics of a CCLA of the next generation (WIN) in combination with different β-titanium archwire dimensions that are routinely used in lingual orthodontic treatment. The results revealed that after an initial stage of torque play, characteristic of each archwire dimension, an almost linear relationship between torque angle and torque moment developed, with the fullsize archwires showing steeper slopes than the undersized study samples.

Prior to a discussion of the findings in comparison to the current literature, some critical considerations of the method should be addressed.

Measuring ten archwires of each dimension did not serve the determination of the error of the method (in that case, one archwire could have been measured ten times in same the experimental set-up) rather than accounting for dimensional variation of each batch of archwires investigated. As this was indeed the case, the torque angle/torque moment curves for ten wires of a supposedly identical dimension showed slight incongruities. However, those were minor and did not affect the overall interpretation of the findings.

In our experimental set-up, the torque moment is not only influenced by the play between a single bracket and the wire, but also by the play of the wire in the neighbouring brackets. This is why a torque moment curve usually shows three characteristic regions with an initial flat part representing the play between the bracket slot and the archwire followed by a first bend with a steeper slope thereafter coinciding with the end of torque play of a single bracket-archwire system [24]. A second bend of the curve indicates the contact of the wire with the slot walls of the neighbouring brackets with a subsequent increase of the torque moment depending on the torsional stiffness of the archwire engaged [14]. Theoretically, these considerations might make the interpretation of the data obtained more difficult, since neighbouring brackets might have varying slot geometries and sizes. However, given the high precision of WIN bracket slots with only one specimen of a recent study sample having a slot size of 0.0181” as opposed to exactly 0.0180” in all the others [23], variance in slot size can be neglected in the discussion of the present findings. Along these lines, the torque angle/torque moment curves for our fullsize archwires do not show those three segments previously described; this is due to the slight oversizing of the 0.018”×0.018” β-titanium wires and the corresponding lack of torque play in the bracket of interest and the two neighbouring brackets as well. Therefore, the first and second characteristic bend of the curve in a three bracket model are missing and only the third stretch is visible. For the undersized archwires analyzed, a characteristic third segment of the curve was not recordable, but might have become visible after the application of higher forces and resulting moments.

A further issue to be kept in mind when comparing the present findings to the literature addresses the absolute numbers for the torque moment which are twice as high in a model with neighbouring brackets by contrast with a one bracket system. As stated at the outset, the type of ligature affects the transmitted torque moment as well. Elastomeric ligatures, as used in our experimental set-up, press the archwire to the bottom of the slot and reduce part of the play. They were found to exhibit a rapid force decay resulting from intraoral moisture and heat sensitivity and from permanent deformation under stretching. Therefore, their use was not recommended in cases where a complete seating of the archwire is required [25]. However, our experiments were conducted under *ex vivo* conditions where only the issue of permanent deformation might be of importance, but, at least for the slot filling archwires, the choice of ligature is neglectable, since there is a press fit between the bracket slot and the archwire due to the manufacturing precision of the components.

As expected, the effective torque play in our study was little for the fullsize archwires and gradually increased with decreasing wire dimension. Surprisingly, the torque play of the 0.018”×0.025” β-titanium wires was slightly higher than for the 0.018”×0.018” specimens, but this observation can be explained by the help of the data on the dimensional precision of the wire batches showing a slight undersizing of the former as opposed to an oversizing of the latter. The absolute numbers for torque play were slightly lower than the figures reported by Daratsianos in his study on Incognito™ brackets (3 M Top-Service, Bad Essen, Germany) which can be explained by the enhanced manufacturing precision of the bracket slot with WIN as compared to Incognito™, the latter ranging from 0.0180” to 0.0183” averaging an overdimensioning of 0.5%. With those minor deviations from the stated values, both, WIN and Incognito™ by far fulfill the requirements for slot precision of the German quality control committee as stated in DIN13971-2, which allows an 0.0180” slot to vary from 0.0180” to 0.0193” [26]. Apart from the disadvantages associated with torque play, there are also situations where a certain play might be favourable, e.g., it facilitates an easier insertion of the archwire when torque control is not crucial and, thus, saving chair time. Moreover, torque play can compensate for anatomical particularities of the individual and for different clinical techniques of the practitioner. Finally, it might make up for imprecision in bracket positioning which is not that much of an issue anymore since the introduction of reliable indirect bonding procedures.

Looking at the findings in greater detail, the torque moment slope for 0.018”×0.025” β-titanium archwires was steeper than for 0.018”×0.018” β-titanium indicating a higher stiffness which is useful in cases where higher moments are needed. However, higher stiffness also means a less constant force over time during deactivation of the appliance and a relative difficulty to apply defined, light optimal forces as a result of the increased load/deflection rate [18,27,28].

The gradual flattening of the slopes with decreasing wire dimension qualifies those undersized archwires for clinical situations where sliding is required, but also indicates that when torque control in the incisor area is crucial, e.g., during the retraction phase in extraction cases, special measures in terms of torque bends have to be taken to accomplish this aim.

A comparison of the torque characteristics for the 0.0175”×0.0175” β-titanium archwire in our experiments with the data reported by Sifakakis *et al*. examining the same material and dimension in combination with an Incognito™ appliance [18] (Figure 8) yields conflicting results for the steepness of the slope as well as for initial torque play, the latter almost lacking in the cited study. This lack of play contrasts with our data where even the batch of supposedly slot filling 0.018”×0.025” β-titanium wires required a minimum of 2° of twist before a measurable effective torque moment developed. However, Sifakakis *et al*. used a different measuring device, namely the orthodontic measurement and simulation system (OMSS) for data acquisition and, therefore, a direct comparison of both data sets might not be possible.

The results of the present investigation provide valuable information on the torque characteristics of different orthodontic wires in a CCLA of the next generation, but a direct correlation of the findings and numbers to a clinical situation should be considered with caution for several reasons. Firstly, we calculated the resulting moments of forces from different angles of twist after activation of the wire. Actually, the deactivation characteristics would be of greater importance for tooth movement clinically, and point to slightly flatter slopes coincident with less torque expression during deactivation due to permanent plastic deformation of the wire, but also of the bracket during activation [29,30]. Additionally, friction and binding as well as bevelling of the edges of the wire and warping of the slot profile have been discussed as explanatory factors [18]. The latter possibility can be ruled out for WIN brackets though, based on own measurements demonstrating the dimensional stability of the slots before and after applying torqueing moments (unpublished data). Second, the force system eventually acting in an oral microenvironment will most likely vary because of the presence of the periodontal ligament whose mechanical characteristics influence the resulting force system. Nevertheless, the precise predictability of the prospective torque moment at a given twist angle with the investigated CCLA is a double-edged sword that represents a powerful tool that can be exploited when needed, but at the same time requires careful treatment planning in order to avoid undesired side effects when considering the periodontal architecture.

## Competing interests

DW is the inventor of the WIN appliance analyzed in the present study.

## Authors’ contributions

DW and RSP suggested the original idea for the paper. CB and DW carried out all of the measurements and analyzed the data. CB and SL made the literature search, and wrote the main part of the manuscript. SL, RSP and DW reviewed and contributed to the writing of all iterations of the paper. All authors approved the final manuscript.
